# Item distribution, internal consistency and structural validity of the German language person-centred climate questionnaire - staff version (PCQ-G-S): a cross-sectional study

**DOI:** 10.1186/s12877-023-04528-3

**Published:** 2024-01-12

**Authors:** Denise Wilfling, Ralph Möhler, Almuth Berg, Jonas Dörner, Natascha Bartmann, Thomas Klatt, Gabriele Meyer, Margareta Halek, Sascha Köpke, Martin N. Dichter

**Affiliations:** 1 Institute of Social Medicine and Epidemiology, Nursing Research Unit, University of Lübeck, Lübeck, Germany; 2 Institute for Health Services Research and Health Economics, Centre for Health and Society, Medical Faculty, University Hospital Düsseldorf, Heinrich-Heine-University Düsseldorf, Düsseldorf, Germany; 3 Institute for Health and Nursing Science, Medical Faculty, Martin Luther University Halle- Wittenberg, Halle (Saale), Germany; 4 Deutsches Zentrum für Neurodegenerative Erkrankungen (DZNE), Witten, Germany; 5 School of Nursing Science, Faculty of Health, Witten/Herdecke University, Witten, Germany; 6 Institute of Nursing Science, Faculty of Medicine, University of Cologne, University Hospital Cologne, Cologne, Germany

**Keywords:** Person-centeredness, Dementia, Validity, Instrument, Psychometric properties, Nursing homes

## Abstract

**Background:**

Person-centredness is considered as best practice for people living with dementia. A frequently used instrument to assess person-centredness of a care environment is the Person-centred Climate Questionnaire (PCQ). The questionnaire comprises of 14 items with the three subscales a climate of safety, a climate of everydayness and a climate of community.

**Aim:**

The aim of the study is to describe the translation process of the English language Person-centred Climate Questionnaire (Staff version, Patient version, Family version) into German language (PCQ-G) and to evaluate the first psychometric properties of the German language Person-centred Climate Questionnaire– Staff version (PCQ-G-S).

**Methods:**

We conducted a cross-sectional study. The three versions of the 14-item English PCQ were translated into German language (PCQ-G) based on the recommendations for cross-cultural adaption of measures. Item distribution, internal consistency and structural validity of the questionnaire were assessed among nursing home staff (PCQ-G-S). Item distribution was calculated using descriptive statistics. Structural validity was tested using principal component analysis (PCA), and internal consistency was assessed for the resulting subscales using Cronbach’s alpha. Data collection took place from May to September 2021.

**Results:**

A total sample of 120 nurses was included in the data analysis. Nine out of 14 items of the PCQ-G-S demonstrated acceptable item difficulty, while five times showed a ceiling effect. The PCA analysis demonstrated a strong structural validity for a three-factor solution explaining 68.6% of the total variance. The three subscales demonstrated a good internal consistency with Cronbach’s alpha scores of 0.8 for each of the subscales.

**Conclusion:**

The analysis of the 14-item German version (PCQ-G-S) showed first evidence for a strong internal consistency and structural validity for evaluating staff perceptions of the person-centredness in German nursing homes. Based on this, further investigations for scale validity of the PCQ-G versions should be carried out.

**Supplementary Information:**

The online version contains supplementary material available at 10.1186/s12877-023-04528-3.

## Background

In Germany, 1.8 million people live with dementia [1] and one third of them live in a long-term care facility [2]. Worldwide, around 57.4 million people are affected, and this number will increase 152.8 million in 2050 [3]. Dementia is a clinical syndrome, characterised by cognitive, neuropsychiatric, and functional symptoms. Psychological and psychiatric changes finally lead to restrictions in daily life [4]. The care of people living with dementia is challenging for all people involved, i.e. the person living with dementia, their family members and health professionals, due to frequently occuring changed behaviour like aggression, agitation, sleep disturbances, wandering and restlessness [5].

In order to meet the complex care needs of people living with dementia, it is necessary to provide care based on patients’ individual needs [6]. Person-centredness is considered as best practice for people living with dementia and essential for high-quality long-term care for older people [7]. Person-centred care (PCC) was developed by Tom Kitwood, based on Roger’s social-psychological theory of personhood [8]. It is based on an established therapeutically relationship between the respective person and the health professional and means respect for the person, the individual’s right to self-determination, mutual respect and understanding [9]. To provide PCC, a supportive care environment is needed. This includes, for example, creating a PCC culture, implementing PCC educational programs for staff or designing health care facilities promoting PCC [10]. For this reason, some organisational conditions are necessary, e.g., PCC skills training for health professionals and creating a person-centred culture and environment. It is essential, that the organisation (e.g., the nursing home) creates conditions to enable person-centredness [11]. It becomes clear, that the implementation of PCC is very complex and this change process is time consuming [10]. In recent years, PCC has become a key indicator of quality in health care. In the course of this, numerous measurement instruments have been developed that capture person-centredness or related constructs [12].

An early developed, theoretically based and in research frequently used instrument is the Person-centred Climate Questionnaire (PCQ) [13–15]. The PCQ was developed based on a theoretical concept regarding supportive care settings [16], literature and a content validity analysis [13]. The original Swedish 14-item version for patients (PCQ-P) was developed and later on supplemented by another version for health care staff (PCQ-S) and family members (PCQ-F). Person-centred care concerns the patient, family and staff, why different scales are needed to address the different perspectives and to assess to what extent family members or health care staff perceive the care environment as person-centred.

The items of these versions are identical, they are answered using a six-point scale (1 = No, I disagree completely to 6 = Yes, I agree completely). Different versions only differ in their perspective. The instrument items operationalise the following subscales: a climate of safety (five items), a climate of everydayness (five items) and a climate of community (four items). All items are sum scored and scores can range from 14 (a climate not very person-centred) to 84 (a climate very person-centred) [17]. After the PCQ-S was translated into English [18], numerous further translations and psychometric evaluation studies were carried out for the Norwegian PCQ-S [19], the Chinese PCQ-S [20], and the Slovenian PCQ-S [21]. A German version of the instrument is not available so far. Therefore, we translated all three versions of the questionnaire into German language within the project “MoNoPol-Sleep - Multi-modal, non-pharmacological intervention to avoid sleep disturbances in people living in nursing home with dementia” [22] and piloted. We report the translation of the English language PCQ and the evaluation of the item distribution, internal consistency and structural validity of the translated German version based on staff ratings (PCQ-G-S) in a nursing home context.

## Methods

### Study design

A cross-sectional study was conducted to determine the item distribution, internal consistency and structural validity of the PCQ-G-S. The investigation of the structural validity was based on an exploratory factor analysis (principal component analysis). This methodical approach was based on the COSMIN standards for test theory studies [23]. The Ethical Committee of the German Society of Nursing Science approved the study protocol for all study centres (no. 20–016).

### Setting and population

Participants were nurses and nursing assistants of the nursing homes enrolled in the MoNoPol-Sleep study (trial registration: ISRCTN36015309) during baseline assessment. Nursing homes were recruited by three regions in Germany (Lübeck: Northern Germany; Halle (Saale): Eastern Germany; Witten: Western Germany). Each region recruited eight nursing homes, first using already existing contacts. Additionally, nursing homes were recruited by means of nursing home registers, information folders, announcements in relevant nursing journals in Germany and the study website (www.monopol-sleep.de). Nursing homes were contacted via phone or email and verbally informed about the aim and content of the study. Nursing homes with at least 50 residents were eligible for inclusion. Nurses and nursing assistants were included if they were working at least three night shifts in the last three months and were contracted for at least part-time (half-a-day). Inclusion criteria and recruitment have been described in detail elsewhere [22].

### Questionnaire translation

All three versions of the PCQ have been translated into German language (PCQ-G) based on four of the five steps recommended for cross-cultural adaption of measures [24] in the preparatory phase of the MoNoPol-sleep study [22]. Each step was documented in a comprehensible manner. The first stage was the forward translation, performing two independent translations from English to German by two different persons. Both translators were native German speakers with excellent English language skills. Next to the translation, it was possible to enter comments on difficulties in wording or other uncertainties. The second stage contained the synthesis of the translations. Translation results were discussed, inconsistencies were reviewed, and a final translation was agreed. Stage three comprised the back translation. The final version from stage two was back translated by two persons independently. The back-translators had English as mother tongue and excellent skills in German language. Both have been involved with translation issues in research before. Also, in this stage it was possible to enter comments on difficulties in wording or other uncertainties. In stage four an expert committee meeting was carried out. All four translators and the coordinator of the translation process (first author of this paper) were involved in this meeting. During the meeting, all versions of the questionnaire were reviewed, and discrepancies discussed until a consensus was reached. This stage was for validity checking to make sure that the translated version of the questionnaire was reflecting the same item content as the original version. After the fourth stage, the whole documentation of the translation process was sent to the author of the original PCQ, and we received permission for the accuracy of the translation. The final version of the PCQ-G is displayed in Table S1. In a final step, based on the available knowledge from the literature and the translation process, a user manual for the German language PCQ was created that is freely available to potential users [25]. We did not perform a pretest as a fifth step as recommended by Beaton et al. (2000) [24]. This was due to the restrictive protective measures for nursing homes and the enormous burden of nurses caused by the COVID-19 pandemic, which made it difficult to access nurses for a pretest at the time of the measurement translation.

### Data collection procedures

The PCQ-G staff version (PCQ-G-S) was part of a 7-page questionnaire measuring nurses’ attitudes regarding the implementation of change processes, person-centredness in care and inter-professional cooperation, as part of the process evaluation in the MoNoPol-Sleep study [22]. Beside the PCQ-G-S, the questionnaire consisted demographic variables. The questionnaire was handed out by the supervising nurse. Participating nurses and nursing assistants received information about the aim and content of the study at the first page of the questionnaire. Furthermore, they received information that informed consent was provided by filling in the questionnaire. Questionnaires were returned by postal mail or personally collected by one researcher in the nursing home. In general, the application of the PCQ-G-S was based on the recommendations by the authors of the original instrument as documented in the German user manual [25].

### Data analysis

Descriptive statistics were calculated for demographic characteristics and item distribution of the PCQ-G-S items. For the item distribution, the cut-off values were set at < 0.8 and > 3.2, based on the recommendations of Bortz & Döring (2006) [26].

In a second step, an explorative factor analysis was performed based on a principal component analysis (PCA). Reasons for conducting the exploratory factor analysis were: no previous knowledge of the factor structure of the PCQ-G S version and the limited sample of nurses available in the Monopol-Sleep study. In addition, the chosen procedure also corresponds to the procedures for the first psychometric evaluation in other countries ( [19–21].

The prerequisites for conducting a PCA were tested [27]: Measure of sample adequacy was performed with the Kaiser-Meyer-Olkin (KMO) criterion. The KMO should be ≥ 0.5. Additionally, the Bartlett’s test for sphericity was conducted. The common significance level of < 0.05 was used for verification of a non-existent item correlation assumed before conducted the component analysis [27]. After, the factor analysis was performed, based on a PCA using an orthogonal rotational procedure (varimax). The factor extraction followed the criteria: (1) eigenvalues > 1 for a factor (Kaiser-Guttman criterion), and (2) scree plot. Missing values were pairwise excluded. The internal consistency of the scale was evaluated by calculating Cronbach’s α coefficients [28]. Data were entered into SPSS v. 22 [29]. Plausibility checks were carried out during data entry. To ensure data quality, all data were checked by a second person.

## Results

### Characteristics of the sample

A total sample of 120 nurses was included in data analysis. The mean age of participants was 40.7 (SD 11.7), with an average working experience in the care of people living with dementia of 14.6 years (SD 10.1). Participants’ demographic characteristics are displayed in Table 1.

**Table 1:** Characteristics of nurses and nursing assistants (Total N = 120)

Nurses and nursing assistants		
Age, years, mean (SD)		40.7 (± 11.7)
Contract hours, number (%)		
	Full time	46 (50.5)
	Part time	45 (49.5)
Years working in elderly care, mean (SD)		14.6 (± 10.1)
Healthcare training, number (%)		
	Elderly care	57 (50)
	Health care nursing	20 (17.5)
	Paediatric nursing	2 (1.8)
	Other*	35 (30.7)

Missing values were pairwise excluded

* e.g. nursing assistant

### Item distribution

The descriptive investigation of the PCQ-G-S showed a balanced distribution (Table 2). The response option “yes, I agree” was used most often whereas the response option “no, I disagree completely” was used least frequently. Distributions of the other response options also varied. Based on the mean values, five items (item 4, item 11, item 12, item 13, item 14) showed a ceiling effect (> 0.8). Missing value analyses demonstrated very low percentages of missing values in general. Only item 3, 5 and 9 of the PCQ-G-S showed a percentage of missing values of 1.7% and items 2, 4, 7 and 11 a percentage of missing values of 0.8%. The reason for this was nurses’ and nursing assistants’ denial to rate.

**Table 2:** Item distribution per item and total score – German version of the PCQ-G-S (N = 120)

Subscales and Items			Response options								
A climate of safety			No, I disagree completely	No, I disagree	No, I partly disagree	Yes, I partly agree	Yes, I agree	Yes, I agree completely	Missing values	Item difficulty	Mean
	1.	A place where I feel welcome	0	2	6	25	58	29	0 (0)	0.77	4.9 (0.9)
	2.	A place where I feel acknowledged as a person	0	1	9	22	52	35	1 (0.8)	0.78	4.9 (0.9)
	3.	A place where I feel I can be myself	1	7	8	28	44	30	2 (1.7)	0.72	4.7 (1.2)
	4.	A place where the patients are in safe hands^a^	0	5	2	23	44	45	1 (0.8)	**0.88**	5.0 (1.0)
	5.	A place where the staff use a language that the patients can understand	0	1	9	26	43	39	2 (1.7)	0.77	4.9 (1.0)
**A climate of everydayness**											
	6.	A place which feels homely even though it is in an institution	1	8	11	37	41	22	0 (0)	0.69	4.5 (1.1)
	7.	A place where there is something nice to look at	2	4	18	42	42	11	1 (0.8)	0.64	4.3 (1.0)
	8.	A place where it is quiet and peaceful	0	6	19	29	53	13	0 (0)	0.68	4.4 (1.0)
	9.	A place where it is possible to get unpleasant thoughts out of your head	3	11	23	36	33	12	2 (1.7)	0.60	4.0 (1.2)
	10.	A place which is neat and clean	2	4	17	27	45	25	0 (0)	0.71	4.5 (1.2)
**A climate of community**											
	11.	A place where it is easy for the patients to keep in contact with their loved ones^a^	0	2	4	25	40	48	1 (0.8)	**0.81**	5.1 (0.9)
	12.	A place where it is easy for the patients to receive visitors^a^	0	3	1	17	47	52	0 (0)	**0.81**	5.2 (0.9)
	13.	A place where it is easy for the patients to talk to the staff^a^	0	0	6	15	37	62	0 (0)	**0.84**	5.3 (0.9)
	14.	A place where the patients have someone to talk to if they so wish^a^	0	3	6	18	42	51	0 (0)	**0.82**	5.1 (1.0)
**Total Score**											66.6 (10.4)

^a^ Item difficulty: Items with floor effects (< 0.2) or ceiling effects (> 0.8) in boldt. Data are the mean (SD) or number (%). Missing values were pairwise excluded

### Structural validity

**Fig. 1:**
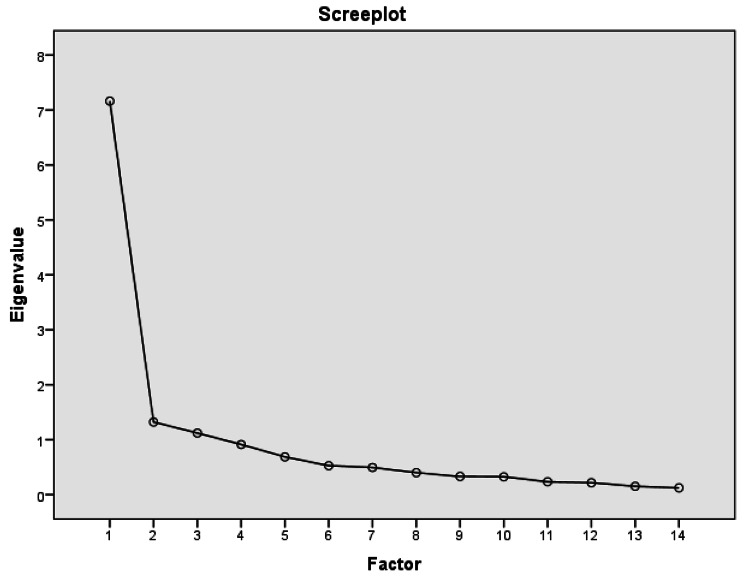
Principal component analysis – scree plot (Total N = 120)

PCA was used to evaluate scale dimensionality and structural validity since Bartlett’s test of sphericity yielded X^2^ = 1048,911, was significant (P < 0.01) and Kaiser-Meyer-Olkin was satisfactory (0.863). This indicates the appropriateness of the factor analysis of the data. Kaiser’s eigenvalue > 1 criterion was used to decide on the number of components to extract and a component loading cut-off of 0.5 was used to conclude if an item loaded on a specific component. Based on a first exploratory PCA, three factors with a Kaiser’s eigenvalue > 1 were determined. The scree plot illustrates the result (see Fig. 1). Thus, the analysis resulted in a 3-component solution, where all 14 items could be assigned with 68.6% of the total variance. The results of the analysis including factor loads of each item are presented in Table 3.

**Table 3:** Structural validity results of the PCQ-G-S based on the total sample (N = 120)

No.	PCQ-G Staff version	Factor 1 A climate of safety	Factor 2 A climate of everydayness	Factor 3 A climate of community
1.	A place where I feel welcome	0.804		
2.	A place where I feel acknowledged as a person	0.793		
3.	A place where I feel I can be myself	0.810		
4.	A place where the patients are in safe hands	0.502		(0.473)
5.	A place where the staff use a language that the patients can understand	0.521		
6.	A place which feels homely even though it is in an institution	(0.470)	0.498	
7.	A place where there is something nice to look at		0.715	
8.	A place where it is quiet and peaceful		0.707	
9.	A place where it is possible to get unpleasant thoughts out of your head	(0.426)	0.725	
10.	A place which is neat and clean		0.805	
11.	A place where it is easy for the patients to keep in contact with their loved ones		(0.472)	0.673
12.	A place where it is easy for the patients to receive visitors		(0.409)	0.779
13.	A place where it is easy for the patients to talk to the staff			0.748
14.	A place where the patients have someone to talk to if they so wish	0.442		0.788
	**Cumulative explained variance (%)**	51,60	61,07	68,88
	**Cronbach’s alpha**	0,845	0,877	0,867
	**Kaiser-Meyer-Olkin (KMO) criterion**	0,863		
	**Bartlett’s test** **of sphericity**	P < 0,005		

Only factor loadings > 0.40 are presented; factor loadings in parentheses imply that a specific item loads to more than one factor and that the factor loading to the other factor is higher

Missing values were pairwise excluded

### Internal consistency

The reliability analysis using Cronbach’s alpha coefficient of the 14-item PCQ-G-S showed a strong internal consistency based on Cronbach’s alpha scores for each of the three subscales: “a climate of safety”: alpha = 0.845, “a climate of everydayness”: alpha = 0.877 and “a climate of community”: alpha = 0.867 (Table 3).

## Discussion

The investigation of the item distribution of the PCQ-G-S demonstrated a balanced distribution of the six response options. Nine out of 14 items showed an acceptable item difficulty, but five items (item 4, 11, 12, 13, 14) showed a ceiling effect (> 0.8). However, it should not be generally indicated to cancel these items of the PCQ-G-S. Instead, it must be considered that this is an exploratory study and further evaluation of the scale validity are needed including a larger sample. Since the item distribution has not yet been examined in other studies, no comparison of the results is possible. Further research is justified needed here, because the identified ceiling effects affect all items of the subscale climate of community and 36% of all PCQ-G-S items.

The results for structural validity show that the original factor structure of the PCQ-G is robust. Similar to the original Swedish version [13], the 14 items of the PCQ-G-S could be assigned to the three subscales a climate of safety, a climate of everydayness, and a climate of community. The same instrument structure was found for the Swedish (Edvardsson et al., 2009), Norwegian [19], Slovenian [21], and Chinese [20] versions. Thus, Cai et al. (2017) found a stable three-factor solution explaining 73.3% of the total variance for the Chinese version (“a climate of safety”: 0.58 to 0.84; “a climate of everydayness”: 0.68 to 0.82”; “a climate of community”: 0.64 to 0.66), Bergland et al. (2012) reported the three-factor solution that explained nearly 68% of the variance in the data for the Norwegian version (“a climate of safety”: 0.55 to 0.84; “a climate of everydayness”: 0.49 to 0.83”; “a climate of community”: 0.62 to 0.80) and Vrbnjak et al. (2017) found a three-factor solution that explained 71.22% of the variance in the data of the Slovenian version (“a climate of safety”: 0.59 to 0.87; “a climate of everydayness”: 0.77 to 0.84”; “a climate of community”: 0.54 to 0.86). The analysis of the original Swedish version resulted in a three-factor solution explaining 60.0% of the total variance (“a climate of safety”: 0.64 to 0.79; “a climate of everydayness”: 0.57 to 0.78, “a climate of community”: 0.58–0.82) [17]. Therefore, scale dimensionality could be seen as confirmed.

Psychometric evaluation of the English PCQ-S resulted in a four-component rotated solution (a climate of safety, a climate of everydayness, a climate of community and a climate of comprehensibility) explaining 71,8% of the total variance [18]. The fourth subscale “a climate of comprehensibility” included four items, relating to the extent staff provided understandable information to patients, patients felt safe, staff were easy to talk to and where patients also had others to talk about their experiences [18]. In the original version, these four items belonged to the subscales a climate of safety and climate of community [17]. Edvardsson et al. (2010) explained the deviation from the original version with three subscales by the fact that the study evaluating the original Swedish version included a sample working on an elective surgery ward with a short length of stay. Because of limited possibility for interactions between staff and patients, the sample in this study may felt prioritising that patients understand implemented medical procedures instead of focusing on proving PCC [18].

Based on a Rasch analysis of the English PCQ-S, residual correlations greater than 0.29 than the mean correlation in the matrix were found. This indicated some evidence of local dependence between two items (item 13 “a place where it is easy for patients to talk to staff”; item 14 “a place where patients have someone to talk”) of subscale three. Since removing or combining item 13 and 14 caused other difficulties, according to Wilberforce et al. (2019) the two items were kept.

The 14-item PCQ-G-S consists of three subscales. It showed strong internal consistency for each of the three subscales a climate of safety (alpha = 0.845), a climate of everydayness (alpha = 0.877), and a climate of community (alpha = 0.867). These results are in line with the results of previous psychometric evaluations. Also, the Swedish [17], English [18], Norwegian [19], Slovenian [21] and Chinese [20] version of the PCQ-S showed internal consistency scores of at least 0.77 for each subscale. Sample sizes in previous studies were comparable to our study. Only in the study of Cai et al. (2017) included more participants (n = 1237).

Although further evaluations in other settings and with lager sample sizes are necessary, e.g. studies evaluation reliability, the PCG-G already contribute to gain a deeper understanding of the extent of person-centred care provided in German-language countries. Additionally, the psychometric properties of the family and patient version should be tested. After that, it would be possible to identify similarities and differences about person-centredness is perceived through patients, families, and staff.

### Strengths and limitations

A major strength of this study is that additionally to the German version of the PCQ, a user manual for the questionnaire (PCQ-G) was developed which is now available online. Thus, an internationally proven questionnaire for the assessment of person-centredness is available for research and practice in the German-speaking countries. Moreover, this is the first study evaluating the psychometric properties of the staff version of the PCQ-G.

This study has some limitations. First, only the staff version of the PCQ-G was evaluated. This means that an evaluation of the patient and family versions is pending and recommended. Second, given the relatively small number of nurses and nursing assistants included in the study, results must be interpreted with caution and have to be proven in a larger study with a confirmatory approach for the PCQ-G-S. Third, the PCQ-G-S was only applied in nursing homes participating in the MoNoPol-Sleep study [22]. Further psychometric validation in different settings is needed to ensure generalisability and to help for further comparisons in different contexts. Fourth, we were unable to perform a pretest of the translated PCG-G-S as recommend by Beaton et al. (2000) [24], because of the restrictions and enormous burden in nursing homes during the COVID-19 pandemic. However, it is crucial to state that no relevant uncertainties regarding the understanding of the items arose in the translation process. Consequently, the decision not to pretest was pragmatic and appropriate considering the context. Moreover, the development of cut-off scores for interpretation purposes is a future goal for the PCQ-G versions.

## Conclusions

The aim of this study was the translation and examination of first psychometric properties of the PCQ-G-S in a nursing home context. The results of this study indicate first evidence for the internal consistency and structural validity for the use of the PCQ-G-S to assess the degree of person-centeredness. Based on these results the questionnaire should be used in further studies to measure person-centredness in nursing homes. Therefore, the item distribution, reliability and especially the construct validity of the PCQ-G-S should be further investigated.

### Electronic supplementary material

Below is the link to the electronic supplementary material.

Supplementary Material 1

## Data Availability

Data is available from the corresponding author on reasonable request.
