# Supplementary material for: Item distribution, internal consistency and structural validity of the German language person-centred climate questionnaire - staff version (PCQ-G-S): a cross-sectional study
Source: BMC Geriatr. 2024 Jan 12;24:57. doi: 10.1186/s12877-023-04528-3 (PMC10787414; doi:10.1186/s12877-023-04528-3)
Supplement: Supplementary file 1 — Supplementary Material 1 [file 12877_2023_4528_MOESM1_ESM.docx]

Table S1: PCQ-G

| Item number | Item content |
| --- | --- |
|  | **Skala 1: Ein Klima von Sicherheit** |
| 1 | Ein Ort, an dem ich mich willkommen fühle. |
| 2 | Ein Ort, an dem ich mich als Person anerkannt fühle. |
| 3 | Ein Ort, an dem ich das Gefühl habe, ich selbst sein zu können. |
| 4 | Ein Ort, an dem die Bewohner:innen in sicheren Händen sind. |
| 5 | Ein Ort, an dem das Personal eine Sprache verwendet, die die Bewohner:innen verstehen können. |
|  | **Skala 2: Ein Klima des Alltagslebens** |
| 6 | Ein Ort, der sich heimisch anfühlt, obwohl er in einer Einrichtung ist. |
| 7 | Ein Ort, an dem es etwas Schönes zu sehen gibt. |
| 8 | Ein Ort, an dem es ruhig und friedlich ist. |
| 9 | Ein Ort, an dem es möglich ist, unangenehme Gedanken aus dem Kopf zu bekommen. |
| 10 | Ein Ort, der ordentlich und sauber ist. |
|  | **Skala 3: Ein Klima von Gemeinschaft** |
| 11 | Ein Ort, an dem es für die Bewohner:innen leicht ist, mit ihren Lieben in Kontakt zu bleiben. |
| 12 | Ein Ort, an dem es für die Bewohner:innen einfach ist, Besuch zu empfangen. |
| 13 | Ein Ort, an dem es für die Bewohner:innen einfach ist, mit dem Personal zu sprechen. |
| 14 | Ein Ort, an dem die Bewohner:innen jemanden haben, mit dem sie reden können, wenn sie möchten. |
